# Diagnostic Model for Discrimination Between Tuberculous Meningitis and Bacterial Meningitis

**DOI:** 10.3389/fimmu.2021.731876

**Published:** 2021-11-12

**Authors:** Ying Luo, Ying Xue, Qun Lin, Liyan Mao, Guoxing Tang, Huijuan Song, Wei Liu, Shiji Wu, Weiyong Liu, Yu Zhou, Lingqing Xu, Zhigang Xiong, Ting Wang, Xu Yuan, Yong Gan, Ziyong Sun, Feng Wang

**Affiliations:** ^1^ Department of Laboratory Medicine, Tongji Hospital, Tongji Medical College, Huazhong University of Science and Technology, Wuhan, China; ^2^ Department of Immunology, School of Basic Medicine, Tongji Medical College, Huazhong University of Science and Technology, Wuhan, China; ^3^ Laboratory Medicine Center, Nanfang Hospital, Southern Medical University, Guangzhou, Guangdong, China; ^4^ Department of Laboratory Medicine, Zhejiang Provincial People’s Hospital, People’s Hospital of Hangzhou Medical College, Hangzhou, China; ^5^ Qingyuan People’s Hospital, The Sixth Affiliated Hospital of Guangzhou Medical University, Qingyuan, China; ^6^ Department of Social Medicine and Health Management, School of Public Health, Tongji Medical College, Huazhong University of Science and Technology, Wuhan, China

**Keywords:** tuberculous meningitis, bacterial meningitis, differential diagnosis, TBAg/PHA ratio, diagnostic model

## Abstract

**Background:**

The differential diagnosis between tuberculous meningitis (TBM) and bacterial meningitis (BM) remains challenging in clinical practice. This study aimed to establish a diagnostic model that could accurately distinguish TBM from BM.

**Methods:**

Patients with TBM or BM were recruited between January 2017 and January 2021 at Tongji Hospital (Qiaokou cohort) and Sino-French New City Hospital (Caidian cohort). The detection for indicators involved in cerebrospinal fluid (CSF) and T-SPOT assay were performed simultaneously. Multivariate logistic regression was used to create a diagnostic model.

**Results:**

A total of 174 patients (76 TBM and 98 BM) and another 105 cases (39 TBM and 66 BM) were enrolled from Qiaokou cohort and Caidian cohort, respectively. Significantly higher level of CSF lymphocyte proportion while significantly lower levels of CSF chlorine, nucleated cell count, and neutrophil proportion were observed in TBM group when comparing with those in BM group. However, receiver operating characteristic (ROC) curve analysis showed that the areas under the ROC curve (AUCs) produced by these indicators were all under 0.8. Meanwhile, tuberculosis-specific antigen/phytohemagglutinin (TBAg/PHA) ratio yielded an AUC of 0.889 (95% CI, 0.840–0.938) in distinguishing TBM from BM, with a sensitivity of 68.42% (95% CI, 57.30%–77.77%) and a specificity of 92.86% (95% CI, 85.98%–96.50%) when a cutoff value of 0.163 was used. Consequently, we successfully established a diagnostic model based on the combination of TBAg/PHA ratio, CSF chlorine, CSF nucleated cell count, and CSF lymphocyte proportion for discrimination between TBM and BM. The established model showed good performance in differentiating TBM from BM (AUC: 0.949; 95% CI, 0.921–0.978), with 81.58% (95% CI, 71.42%–88.70%) sensitivity and 91.84% (95% CI, 84.71%–95.81%) specificity. The performance of the diagnostic model obtained in Qiaokou cohort was further validated in Caidian cohort. The diagnostic model in Caidian cohort produced an AUC of 0.923 (95% CI, 0.867–0.980) with 79.49% (95% CI, 64.47%–89.22%) sensitivity and 90.91% (95% CI, 81.55%–95.77%) specificity.

**Conclusions:**

The diagnostic model established based on the combination of four indicators had excellent utility in the discrimination between TBM and BM.

## Introduction

Tuberculosis (TB), caused by *Mycobacterium tuberculosis* (MTB) infection, remains an ongoing and predominant health issue worldwide, with an estimated 10 million incident cases and 1.4 million deaths in 2019 globally (1). China is a high-TB-burden country with an average of 58 cases per 100,000 subjects (1). It was estimated that there were approximately 833, 000 cases in 2019 (1). Tuberculous meningitis (TBM), the most severe type of extrapulmonary TB, is prevalent in countries with high TB burden (2–4). Given that the manifestations of TBM can mimic other infectious diseases such as bacterial meningitis (BM), it is difficult for clinicians to diagnose TBM. Meanwhile, accurate and in-time diagnosis of TBM is critical for effective treatment. Consequently, there is an urgent need for developing accurate and rapid approaches for TBM diagnosis.

Unfortunately, the current diagnostic approaches for TBM have either low sensitivity (smear microscopy) or are time-consuming (mycobacterial culture) (5). Detection of MTB DNA using molecular technologies such GeneXpert MTB/RIF and GeneXpert MTB/RIF Ultra provides quicker results than mycobacterial culture, yet more than half of cases cannot be bacteriologically confirmed (6). Specially, microscopy is rapid and widely available but could only detect 10%–20% cases (7–9). Mycobacterial culture and GeneXpert MTB/RIF have similar sensitivity of approximately 40% (6). High level of cerebrospinal fluid (CSF) adenosine deaminase was found in patients with TBM due to the activation of T lymphocytes in response to TB antigens (10). However, the sensitivity of the indicator is variable, and false-positive results were commonly noted in patients with HIV infection (10–12). Besides, T-SPOT.TB (T-SPOT), one of two commercially available interferon-gamma release assays, has been broadly used for diagnosis of MTB infection. Nevertheless, a recent meta-analysis conducted by Luo et al. (13) revealed a pooled sensitivity of 78% and specificity of 68% using peripheral blood T-SPOT and a pooled sensitivity of 76% and specificity of 88% using CSF T-SPOT in the diagnosis of TBM. Beyond that, indeterminate results are commonly noted, as sufficient sample volumes are usually unavailable, further limiting the use of CSF T-SPOT in clinical practice (14).

Given that microbiological confirmation is achieved in less than half of all cases due to the paucibacillary nature of the disease, the diagnosis of TBM has to rely on clinical, epidemiological, radiological, and routine laboratory features of patients. Consistent with this notion, the combination of different parameters might offer increased sensitivity and specificity over conventional approaches based on only one marker (15). As expected, some studies have tried to establish the diagnostic models targeting TBM diagnosis. For example, Yang et al. (16) found a four-parameter signature that could discriminate TBM from BM with a sensitivity of 98% and a specificity of 82%. Another clinical prediction rule established by Vibha et al. (17) showed a sensitivity of 95.71% and a specificity of 97.63% in distinguishing TBM from BM. However, few models were validated in an independent cohort. Surprisingly, few studies incorporated T-SPOT assay into diagnostic models, while this assay has been shown to have some potential in the diagnosis of TBM (18, 19). In addition, our previous studies have revealed that TB-specific antigen/phytohemagglutinin (TBAg/PHA) ratio of T-SPOT displayed improved performance on the diagnosis of extrapulmonary TB compared to directly using T-SPOT antigen spot numbers (20). This evidence suggested that the TBAg/PHA ratio may be an important laboratory marker that could be incorporated into TBM diagnostic model. In the present study, we attempted to establish a diagnostic model that had a prominent effect in discriminating TBM from BM.

## Methods

### Study Design

The present study was conducted between January 2017 and January 2021. Adult patients over 17 years of age with TBM or BM were consecutively recruited from Tongji Hospital (Qiaokou cohort, the largest hospital in central China) and Sino-French New City Hospital (Caidian cohort, a branch hospital of Tongji Hospital). We evaluated the two datasets separately for training (Qiaokou cohort) and validation (Caidian cohort). TBM was diagnosed by positive MTB culture (Mycobacterial Growth Indicator Tube 960 and Lowenstein–Jensen media) and/or positive GeneXpert MTB/RIF (Cepheid, Sunnyvale, CA, USA) in CSF, with clinical symptoms and radiological characteristics suggestive of TBM (21–23). BM was diagnosed when pathogenic bacteria were isolated from the CSF using culture (24, 25). Matrix-assisted laser desorption/ionization time-of-flight mass spectrometry (MALDI-TOF-MS) was used for bacteria identification. Since only patients with confirmed diagnosis were included in the current study, those who were unable to identify due to insufficient evidence or report missingness were excluded from the analysis. T-SPOT assay and other routine laboratory tests such as CSF cytology and biochemistry were performed simultaneously. Patients who received anti-TB treatment within 2 weeks prior to enrollment were excluded. The study was approved by the Ethics Committee of Tongji Hospital, Tongji Medical College, Huazhong University of Science and Technology.

### T-SPOT Assay on Peripheral Blood

Heparinized peripheral blood was collected and analyzed using T-SPOT assay (Oxford Immunotec, Oxford, UK) according to the manufacturer’s instructions. Briefly, the isolated peripheral blood mononuclear cells (PBMCs) (2.5 × 10^5^) were added to 96-well plates precoated with anti-interferon-gamma (anti-IFN-γ) antibody. Four wells were used for each subject: medium well, PHA well, early secreted antigenic target 6 (ESAT-6), and culture filtrate protein 10 (CFP-10) wells. Plates were incubated for 16–20 h at 37°C with 5% CO_2_ and developed using an anti-IFN-γ antibody conjugate and substrate to detect the presence of secreted IFN-γ. Spot-forming cells (SFCs) were counted with an automated ELISPOT reader (CTL Analyzers, Cleveland, OH, USA). The test result was positive if ESAT-6 and/or CFP-10 minus negative control ≥6 spots. The test result was negative if both ESAT-6 minus negative control and CFP-10 minus negative control ≤5 spots. Results were considered undetermined if the spot amounts in the PHA well were <20 or if spot amounts in the medium well were >10. We calculated the ratios of 1) ESAT-6 SFC to PHA SFC and 2) CFP-10 SFC to PHA SFC. The larger of the above two values was defined as the TBAg/PHA ratio of one participant (26).

### Statistical Analysis

Continuous variables were expressed as means ± standards deviation (SD) or median (interquartile range). Categorical variables were expressed as number (%). Comparison was performed using Mann–Whitney *U* test for continuous variables and chi-square test or Fisher’s exact test for categorical variables. All statistical tests were two-sided. Statistical significance was considered when *P* < 0.05. In order to develop a diagnostic model, all variables with statistical significance were taken as candidates for multivariable logistic regression analyses, and the regression equation (diagnostic model) was obtained. The regression coefficients of the model were regarded as the weights for the respective variables, and a score for each patient was calculated. The performance of diagnostic models was evaluated by the receiver operating characteristic (ROC) curve analysis. Sensitivity, specificity, positive predictive value (PPV), negative predictive value (NPV), positive likelihood ratio (PLR), negative likelihood ratio (NLR), and accuracy, together with their 95% confidence intervals (CI), were calculated. Data were analyzed using SPSS version 25.0 (SPSS, Inc., Chicago, IL, USA), GraphPad Prism version 8 (GraphPad Software, San Diego, CA, USA), and R 4.0.2 program (R Core Team).

## Results

### Clinical and Demographic Characteristics of the Included Participants

A total of 174 patients, including 76 TBM and 98 BM, were enrolled in Qiaokou cohort. Another 105 subjects that consisted of 39 TBM and 66 BM were included in Caidian cohort. The main clinical and demographic data of the patients were summarized in **Table 1**. There was no statistical difference between TBM group and BM group in age and gender among these two cohorts. Headache and fever were the most common symptoms in the two groups. There was no statistical difference in underlying condition or illness between TBM and BM. The etiology information for BM was presented in **Supplementary Table S1**.

**Table 1:** Demographic and clinical characteristics of recruited participants.

Variables	Qiaokou cohort (training set)		P*	Caidian cohort (validation set)		P*	*P* ^†^
	TBM (n=76)	BM (n=98)		TBM (n=39)	BM (n=66)		
Age, years	44 (27-58)	42 (30-54)	0.926	45 (29-59)	39 (26-54)	0.294	0.689
Gender, male	51 (67.11%)	69 (70.41%)	0.64	26 (66.67%)	43 (65.15%)	0.874	0.574
Symptoms							
Headache	62 (81.58%)	70 (71.43%)	0.121	30 (76.92%)	47 (71.21%)	0.523	0.637
Fever	50 (65.79%)	53 (54.08%)	0.119	25 (64.1%)	37 (56.06%)	0.418	0.981
Vomiting	26 (34.21%)	37 (37.76%)	0.629	16 (41.03%)	25 (37.88%)	0.749	0.634
Convulsion	6 (7.89%)	9 (9.18%)	0.764	4 (10.26%)	5 (7.58%)	0.91	0.989
Irritability	4 (5.26%)	8 (8.16%)	0.454	3 (7.69%)	4 (6.06%)	0.935	0.941
Weight loss	7 (9.21%)	7 (7.14%)	0.619	3 (7.69%)	7 (10.61%)	0.883	0.670
Underlying condition or illness							
Diabetes mellitus	5 (6.58%)	10 (10.2%)	0.398	4 (10.26%)	6 (9.09%)	0.883	0.798
Hypertension	3 (3.95%)	9 (9.18%)	0.176	5 (12.82%)	3 (4.55%)	0.245	0.821
Solid tumor	9 (11.84%)	8 (8.16%)	0.418	4 (10.26%)	10 (15.15%)	0.476	0.359
Hematological malignancy	6 (7.89%)	16 (16.33%)	0.097	3 (7.69%)	8 (12.12%)	0.699	0.587
Nephritis or kidney failure	5 (6.58%)	5 (5.1%)	0.931	5 (12.82%)	4 (6.06%)	0.404	0.364
Virus hepatitis or cirrhosis	10 (13.16%)	12 (12.24%)	0.857	6 (15.38%)	8 (12.12%)	0.635	0.868
Heart disease	6 (7.89%)	8 (8.16%)	0.949	5 (12.82%)	6 (9.09%)	0.785	0.491
Organ transplantation	2 (2.63%)	3 (3.06%)	0.772	1 (2.56%)	3 (4.55%)	0.988	0.937
HIV infection	2 (2.63%)	0 (0%)	0.189	2 (5.13%)	1 (1.52%)	0.64	0.565
Immunosuppressive condition^‡^	10 (13.16%)	15 (15.31%)	0.689	6 (15.38%)	12 (18.18%)	0.713	0.534
T-SPOT positivity	65 (85.53%)	18 (18.37%)	<0.001	31 (79.49%)	15 (22.73%)	<0.001	0.528

TBM, tuberculous meningitis; BM, bacterial meningitis. *Comparisons were performed between TBM group and BM group using Mann-Whitney U test, Chi-square test, or Fisher’s exact test. ^†^Comparisons were performed between Qiaokou and Caidian cohorts using Mann-Whitney U test, Chi-square test, or Fisher’s exact test. ^‡^Patients who underwent chemotherapy or took immunosuppressants within 3 months. Data are presented as number (percentage) or medians (25th - 75th centiles).

### Performance of Cerebrospinal Fluid Indicators for Differentiating Tuberculous Meningitis From Bacterial Meningitis

**Figure 1:**
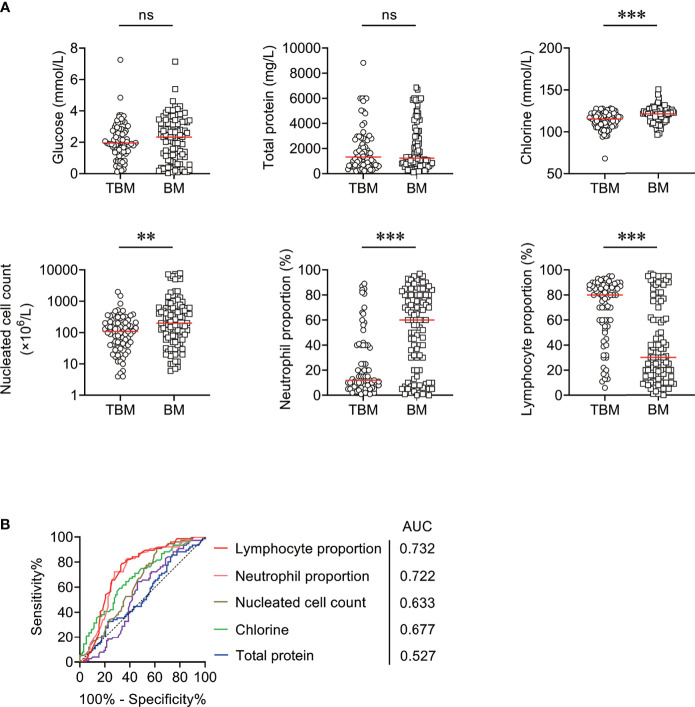
The performance of CSF biochemical and cytological indicators for distinguishing TBM from BM in Qiaokou cohort. **(A)** Scatter plots showing the levels of CSF indicators including glucose, total protein, chlorine, nucleated cell count, neutrophil proportion, and lymphocyte proportion in TBM patients (n = 76) and BM patients (n = 98). Horizontal lines indicate the medians. **P < 0.01; ***P < 0.001; ns, no significance (Mann–Whitney *U* test). **(B)** ROC curve analysis showing the performance of CSF indicators in distinguishing TBM from BM. TBM, tuberculous meningitis; BM, bacterial meningitis; ROC, receiver operating characteristic; CSF, cerebrospinal fluid; AUC, area under the ROC curve.

The comparison between TBM group and BM group in CSF biochemical and cytological indicators was performed. It was observed that the levels of CSF chlorine, CSF nucleated cell count, and CSF neutrophil proportion were significantly lower in TBM group than those in BM group (**Figure 1A**). On the contrary, a significantly higher level of CSF lymphocyte proportion was noted in TBM group in comparison with that in BM group (**Figure 1A**). However, there was no statistical difference in the concentration of CSF glucose or total protein between TBM and BM patients (**Figure 1A**). ROC curve analysis was applied to evaluate the discriminatory performance of these indicators. It was found that CSF lymphocyte proportion produced an AUC of 0.732 (95% CI, 0.656–0.808) while CSF neutrophil proportion yielded an AUC of 0.722 (95% CI, 0.645–0.799) in distinguishing TBM from BM (**Table 2** and **Figure 1B**).

**Table 2:** The performance of CSF indicators for differentiating TBM from BM.

Indicators	Cutoff value	AUC (95% CI)	Sensitivity (95% CI)	Specificity (95% CI)	PPV (95% CI)	NPV (95% CI)	PLR (95% CI)	NLR (95% CI)	Accuracy
CSF chlorine (mmol/L)	113	0.677 (0.598–0.756)	40.79% (30.44%–52.02%)	80.61% (71.69%–87.22%)	62.00% (48.15%–74.14%)	63.71% (54.95%–71.64%)	2.1 (1.29–3.42)	0.73 (0.6–0.91)	63.22%
CSF nucleated cell count (×10^6^/L)	75	0.633 (0.551–0.715)	38.16% (28.06%–49.40%)	69.39% (59.68%–77.64%)	49.15% (36.84%–61.57%)	59.13% (49.99%–67.68%)	1.25 (0.82–1.88)	0.89 (0.72–1.11)	55.75%
CSF neutrophil proportion (%)	26	0.722 (0.645–0.799)	72.37% (61.42%–81.16%)	72.45% (62.88%–80.32%)	67.07% (56.34%–76.28%)	77.17% (67.61%–84.56%)	2.63 (1.85–3.73)	0.38 (0.26–0.56)	72.41%
CSF lymphocyte proportion (%)	50	0.732 (0.656–0.808)	78.95% (68.50%–86.61%)	66.33% (56.51%–74.91%)	64.52% (54.39%–73.49%)	80.25% (70.30%–87.46%)	2.34 (1.73–3.17)	0.32 (0.2–0.5)	71.84%

TBM, tuberculous meningitis; BM, bacterial meningitis; AUC, area under the curve; PPV, positive predictive value; NPV, negative predictive value; PLR, positive likelihood ratio; NLR, negative likelihood ratio; CI, confidence interval; CSF, cerebrospinal fluid.

### Diagnostic Value of Tuberculosis-Specific Antigen/Phytohemagglutinin Ratio for Distinguishing Tuberculous Meningitis From Bacterial Meningitis

**Figure 2:**
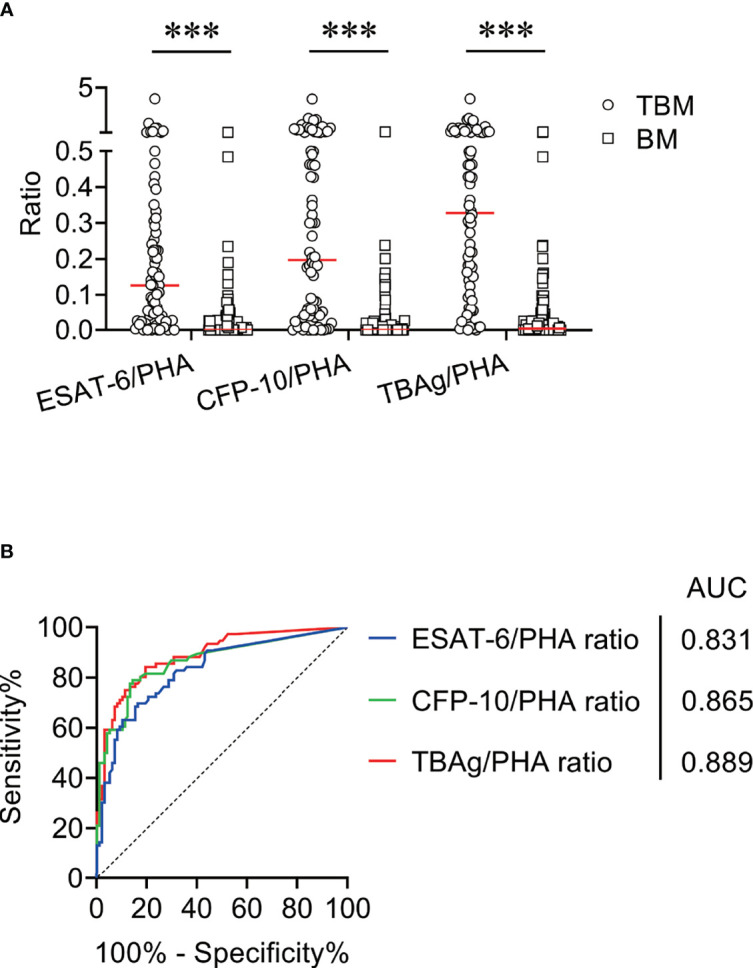
The performance of TBAg/PHA ratio for distinguishing TBM from BM in Qiaokou cohort. **(A)** Scatter plots showing the levels of ESAT-6/PHA ratio, CFP-10/PHA ratio, and TBAg/PHA ratio in TBM patients (n = 76) and BM patients (n = 98). Horizontal lines indicate the medians. ***P < 0.001 (Mann–Whitney *U* test). **(B)** ROC curve analysis showing the performance of ESAT-6/PHA ratio, CFP-10/PHA ratio, and TBAg/PHA ratio in distinguishing TBM from BM. TBM, tuberculous meningitis; BM, bacterial meningitis; ESAT-6, early secreted antigenic target 6; CFP-10, culture filtrate protein 10; PHA, phytohemagglutinin; TBAg, tuberculosis-specific antigen; ROC, receiver operating characteristic; AUC, area under the ROC curve.

We compared ESAT-6/PHA ratio, CFP-10/PHA ratio, and TBAg/PHA ratio between TBM patients and BM patients. It was found that ESAT-6/PHA ratio, CFP-10/PHA ratio, and TBAg/PHA ratio were significantly higher in TBM patients than those in BM patients (**Figure 2A**). ROC curve analysis demonstrated the AUC of ESAT-6/PHA ratio in discriminating TBM from BM to be 0.831 (95% CI, 0.769–0.893). The cutoff value of 0.095 showed a sensitivity of 55.26% (95% CI, 44.10%–65.92%) and specificity of 91.84% (95% CI, 84.71%–95.81%) (**Table 3** and **Figure 2B** **)**. The AUC of CFP-10/PHA ratio in discriminating TBM from BM was 0.865 (95% CI, 0.808–0.922). The sensitivity and specificity were 59.21% (95% CI, 47.98%–69.56%) and 90.82% (95% CI, 83.46%–95.09%), respectively, with the threshold 0.11 (**Table 3** and **Figure 2B** **)**. Furthermore, the discriminatory power measured by the AUC for TBAg/PHA ratio was 0.889 (95% CI, 0.840–0.938). When 0.163 was set as the cutoff value, the sensitivity and specificity were 68.42% (95% CI, 57.30%–77.77%) and 92.86% (95% CI, 85.98%–96.50%), respectively (**Table 3** and **Figure 2B** **)**.

**Table 3:** The performance of TBAg/PHA ratio for distinguishing between TBM and BM.

Indicators	Cutoff value	AUC (95% CI)	Sensitivity (95% CI)	Specificity (95% CI)	PPV (95% CI)	NPV (95% CI)	PLR (95% CI)	NLR (95% CI)	Accuracy
ESAT-6/PHA ratio	0.095	0.831 (0.769–0.893)	55.26% (44.10%–65.92%)	91.84% (84.71%–95.81%)	84.00% (71.49%–91.66%)	72.58% (64.14%–79.67%)	6.77 (3.38–13.55)	0.49 (0.38–0.63)	75.86%
CFP-10/PHA ratio	0.11	0.865 (0.808–0.922)	59.21% (47.98%–69.56%)	90.82% (83.46%–95.09%)	83.33% (71.26%–90.98%)	74.17% (65.67%–81.16%)	6.45 (3.37–12.35)	0.45 (0.34–0.59)	77.01%
TBAg/PHA ratio	0.163	0.889 (0.840–0.938)	68.42% (57.30%–77.77%)	92.86% (85.98%–96.50%)	88.14% (77.48%–94.13%)	79.13% (70.82%–85.56%)	9.58 (4.62–19.88)	0.34 (0.24–0.48)	82.18%

TBAg, tuberculosis antigen; PHA, phytohemagglutinin; TBM, tuberculous meningitis; BM, bacterial meningitis; AUC, area under the curve; PPV, positive predictive value; NPV, negative predictive value; PLR, positive likelihood ratio; NLR, negative likelihood ratio; CI, confidence interval; ESAT-6, early secreted antigenic target 6; CFP-10, culture filtrate protein 10.

### Establishing the Diagnostic Model in Discriminating Tuberculous Meningitis From Bacterial Meningitis

**Figure 3:**
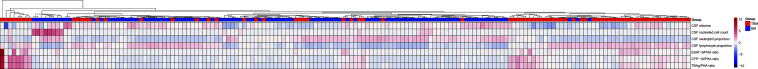
The cluster analysis of various indicators in TBM group and BM group. Heatmap showing the cluster analysis of CSF chlorine, CSF nucleated cell count, CSF neutrophil proportion, CSF lymphocyte proportion, ESAT-6/PHA ratio, CFP-10/PHA ratio, and TBAg/PHA ratio in TBM patients (n = 76) and BM patients (n = 98). Each rectangle indicates a result of a patient. TBM, tuberculous meningitis; BM, bacterial meningitis; CSF, cerebrospinal fluid; ESAT-6, early secreted antigenic target 6; CFP-10, culture filtrate protein 10; PHA, phytohemagglutinin; TBAg, tuberculosis-specific antigen.

**Figure 4:**
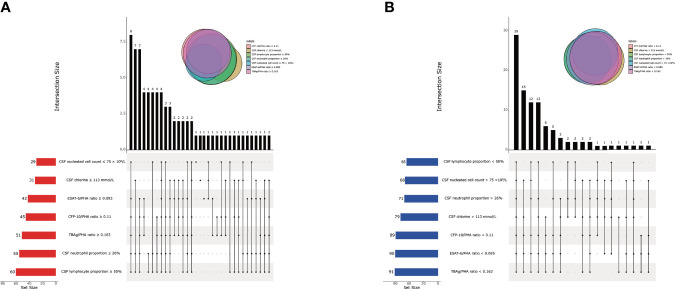
The cross set between various indicators in TBM group and BM group. **(A)** Upset plot showing the cross set between various indicators in TBM group. **(B)** Upset plot showing the cross set between various indicators in BM group. TBM, tuberculous meningitis; BM, bacterial meningitis; CSF, cerebrospinal fluid; ESAT-6, early secreted antigenic target 6; CFP-10, culture filtrate protein 10; PHA, phytohemagglutinin; TBAg, tuberculosis-specific antigen.

**Figure 5:**
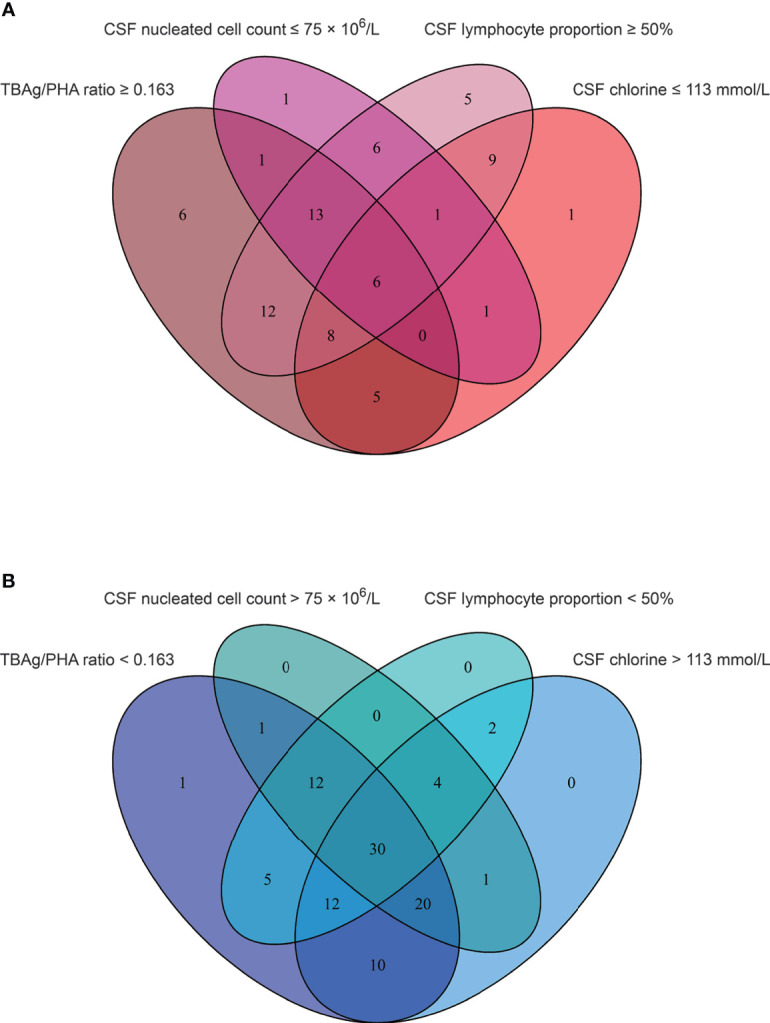
The cross set between various indicators in TBM group and BM group. **(A)** Venn diagrams showing the overlap of TBAg/PHA ratio, CSF nucleated cell count, CSF lymphocyte proportion, and CSF chlorine in TBM patients. **(B)** Venn diagrams showing the overlap of TBAg/PHA ratio, CSF nucleated cell count, CSF lymphocyte proportion, and CSF chlorine in BM patients. TBM, tuberculous meningitis; BM, bacterial meningitis; TBAg, tuberculosis-specific antigen; PHA, phytohemagglutinin; CSF, cerebrospinal fluid.

**Figure 6:**
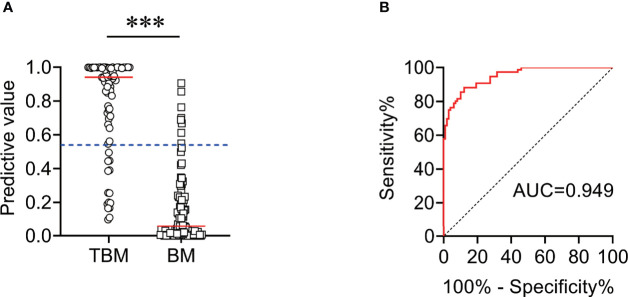
The performance of diagnostic model for discriminating TBM from BM in Qiaokou cohort. **(A)** Scatter plots showing the predictive value of the diagnostic model in TBM patients (n = 76) and BM patients (n = 98). Horizontal lines indicate the medians. ***P < 0.001 (Mann–Whitney *U* test). Blue dotted lines indicate the cutoff value in distinguishing these two groups. **(B)** ROC curve analysis showing the performance of the diagnostic model in distinguishing TBM from BM. ROC, receiver operating characteristic; AUC, area under the ROC curve.

We found that CSF biochemical and cytological indicators as well as TBAg/PHA ratio exhibited either limited or moderate value in discrimination between TBM and BM. In order to identify the possibility of combining CSF indicators and TBAg/PHA ratio to distinguish TBM from BM, we performed heatmap analysis and discovered the potential of combination of these indexes to differentiate TBM from BM (**Figure 3**). Next, we analyzed the cross set of indicators with significant differences in two groups. The overlap of seven indicators indicated the possible conjunct use for stratification (**Figure 4**). To establish the diagnostic model based on a combination of various indicators for distinguishing TBM from BM, all variables with statistical significance were used for multivariable logistic regression analysis. The diagnostic model was built as follows: P = 1/[1 + e^-(11.512*TBAg/PHA ratio - 0.149*CSF chlorine - 0.001*CSF nucleated cell count + 0.041*CSF lymphocyte proportion + 13.677)^]. P, predictive value; e, natural logarithm. Venn diagram showed the overlap of these four parameters in TBM and BM groups and confirmed their appropriate combination (**Figure 5**). The model presented an AUC of 0.949 (95% CI, 0.921–0.978) with a sensitivity of 81.58% (95% CI, 71.42%–88.70%) and specificity of 91.84% (95% CI, 84.71%–95.81%) when 0.54 was used as the cutoff value (**Table 4** and **Figure 6**).

**Table 4:** The performance of diagnostic model for discriminating TBM from BM.

Parameters	Training set	Validation set
Cutoff value	0.54	0.54
AUC (95% CI)	0.949 (0.921–0.978)	0.923 (0.867–0.980)
Sensitivity (95% CI)	81.58% (71.42%–88.70%)	79.49% (64.47%–89.22%)
Specificity (95% CI)	91.84% (84.71%–95.81%)	90.91% (81.55%–95.77%)
PPV (95% CI)	88.57% (79.04%–94.09%)	83.78% (68.86%–92.35%)
NPV (95% CI)	86.54% (78.67%–91.81%)	88.24% (78.47%–93.92%)
PLR (95% CI)	9.99 (5.1–19.58)	8.74 (4.01–19.06)
NLR (95% CI)	0.2 (0.12–0.32)	0.23 (0.12–0.42)
Accuracy	87.36%	86.67%

TBM, tuberculous meningitis; BM, bacterial meningitis; AUC, area under the curve; PPV, positive predictive value; NPV, negative predictive value; PLR, positive likelihood ratio; NLR, negative likelihood ratio; CI, confidence interval.

### Validation of Established Diagnostic Model by an Independent Cohort

**Figure 7:**
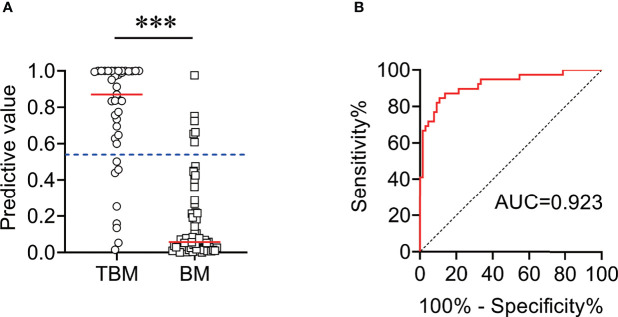
The performance of diagnostic model for discriminating TBM from BM in Caidian cohort. **(A)** Scatter plots showing the predictive value of the diagnostic model in TBM patients (n = 39) and BM patients (n = 66). Horizontal lines indicate the medians. ***P < 0.001 (Mann–Whitney *U* test). Blue dotted lines indicate the cutoff value in distinguishing these two groups. **(B)** ROC curve analysis showing the performance of the diagnostic model in distinguishing TBM from BM. ROC, receiver operating characteristic; AUC, area under the ROC curve.

Another independent set (Caidian cohort) was included to validate our established model. Similar to the training cohort, the diagnostic model could effectively discriminate TBM from BM in the validation cohort. According to the cutoff value obtained in the training cohort, the diagnostic model showed an accuracy of 86.67%, with a sensitivity of 79.49% (95% CI, 64.47%–89.22%) and a specificity of 90.91% (95% CI, 81.55%–95.77%) in discriminating TBM from BM (**Table 4** and **Figure 7**).

## Discussion

Discrimination between TBM and BM remains a challenge in clinical practice. The current bacteriological tests had low sensitivity for the diagnosis of TBM (27, 28). Besides, the lack of accessible and timely methods also contributed to a delay in diagnosis and subsequent morbidity and mortality for TBM patients, particularly those in resource-limited settings (29). Although the potential biomarkers of proteome (30), metabolome (31), and transcriptome (32) have been identified for TBM diagnosis in recent years, these markers have not been sufficiently verified. In this study, we compared the laboratory features including CSF indexes and TBAg/PHA ratio in T-SPOT assay among patients with these two types of meningitis. Importantly, we successfully established and verified a diagnostic model with good performance for distinguishing TBM from BM.

One of the most important reasons why TBM diagnosis is difficult is that the positive rate of microbiological detection methods was low in CSF. It is generally viewed that the sensitivity of acid-fast stain, MTB culture, and GeneXpert MTB/RIF in patients with TBM was much lower than that in patients with pulmonary TB (33–36). It is because that the concentration of MTB pathogen is low in CSF when TBM occurs. Thus, the diagnosis of TBM based on the combination of routine laboratory features is necessary at present. Sometimes, clinicians had to prescribe diagnostic anti-TB therapy to help TBM diagnosis, and missed diagnosis and misdiagnosis are inevitable in real clinical practice. The present study built a four-indicator diagnostic model based on combining CSF indexes and TBAg/PHA ratio. Notably, this four-indicator model had a prominent effect on differentiating TBM from BM, yielding an accuracy of more than 85%, with the AUC more than 0.9. Hereafter, the performance of the model was successfully verified in an independent cohort, further supporting the evidence that the model may have wide applicability in TBM diagnosis in real clinical practice.

It is noteworthy that rare studies had included T-SPOT assay to build a diagnostic model previously. We speculated the reason for this may be that the operating procedures of T-SPOT assay are complicated, and it is generally viewed that the repeatability and reproducibility of the method need to be further verified. However, we have spent a lot of effort in the standardization of T-SPOT assay. We have first emphasized that the substrate incubation time, peripheral blood mononuclear cell counting, and the setting of ELISPOT reader parameters (such as exposure time and sensitivity) are the key factors affecting SFC results of antigen and PHA in T-SPOT assay (20, 37). In addition, we first put forward the concept of TBAg/PHA ratio and found that the performance of TBAg/PHA ratio is better than directly using T-SPOT antigen results not only in distinguishing active tuberculosis (ATB) from latent tuberculosis infection (LTBI) but also in differential diagnosis of pulmonary and extrapulmonary TB (20, 26, 37–41). We also first put forward that 1 × 10^5^ was the optimal number of pleural fluid mononuclear cells for performing pleural fluid T-SPOT and that the mean spot size of ESAT-6 has an adjunctive role in the diagnosis of ATB (42, 43). Thus, our previously published findings suggest that we have accumulated excellent experience in performing T-SPOT assay. To the best of our knowledge, this is the first study to establish the diagnostic model based on the combination of TBAg/PHA ratio and other indicators to discriminate TBM from BM. In view of the fact that TBAg/PHA ratio is a further calculation of T-SPOT results, the model does not increase additional costs.

Several limitations should be noticed in our study. First, the sample size in each cohort, especially the validation cohort, was not large enough. Additional studies including larger cases are required to determine the performance of the established model. Second, the patient population in the current study may not be representative of patients with meningitis in general. In clinical practice, meningitis can have a wider range of causes including viral and fungal infection, as well as autoimmune diseases. Patients with these conditions were not included in this study. Therefore, further investigation with the inclusion of these populations is needed to determine the real utility of the established model. Third, to ensure the reliability of inclusion, our study only included patients with microbiologically diagnosed TBM. However, the diagnosis for clinically identified TBM (probable or possible TBM) was more crucial (6, 44). The validation of the established model in diagnosing probable or possible TBM is also warranted in the future. Fourth, it is important to note that the cost-effectiveness of the application of the models in real-world clinical settings should be evaluated in the future. Finally, it is undeniable that the combination of various measurements not only increases additional manpower, material resources, and costs but also increases the complications and difficulties in the implementation. However, the reality at present is that new technologies have not been fully proven effective and traditional technologies cannot meet clinical needs; the obstacles brought by the joint applications are worth to overcome.

Collectively, our study uncovered and established a novel diagnostic model based on the combination of four indicators with excellent utility in distinguishing TBM from BM. Moreover, our model would have potential in facilitating timely initiation of anti-tuberculosis treatment and improving patients’ outcome.

## Data Availability Statement

The original contributions presented in the study are included in the article/**Supplementary Material**. Further inquiries can be directed to the corresponding authors.

## Ethics Statement

The studies involving human participants were reviewed and approved by the Ethics Committee of Tongji Hospital, Tongji Medical College, Huazhong University of Science and Technology. The patients/participants provided their written informed consent to participate in this study.

## Author Contributions

YL had the original idea of the study. YL conceived and designed the research. YL interpreted data and wrote the article. YL, YX, QL, LM, GT, HS, and WL contributed to the acquisition of clinical data. YL, SW, WYL, YZ, LX, ZX, TW, and XY recruited the participants, performed experiments, and analyzed data. YL, YG, FW, and ZS contributed to the revision of the article. All authors contributed to the article and approved the submitted version.

## Funding

This work was supported by grants from the Fundamental Research Funds for the Central Universities (no. 2021yjsCXCY088), the Special Foundation for National Science and Technology Basic Research Program of China (no. 2019FY101206), the National Natural Science Foundation (no. 81902132), and the Qingyuan People’s Hospital Medical Scientific Research Fund Project (no. 20190209).

## Conflict of Interest

The authors declare that the research was conducted in the absence of any commercial or financial relationships that could be construed as a potential conflict of interest.

## Publisher’s Note

All claims expressed in this article are solely those of the authors and do not necessarily represent those of their affiliated organizations, or those of the publisher, the editors and the reviewers. Any product that may be evaluated in this article, or claim that may be made by its manufacturer, is not guaranteed or endorsed by the publisher.
