# Supplementary material for: Diagnostic Model for Discrimination Between Tuberculous Meningitis and Bacterial Meningitis
Source: Front Immunol. 2021 Nov 12;12:731876. doi: 10.3389/fimmu.2021.731876 (PMC8632769; doi:10.3389/fimmu.2021.731876)
Supplement: Supplementary file 1 [file Table_1.docx]

| **Supplementary Table 1. The etiology information for bacterial meningitis** | | |
| --- | --- | --- |
| Strain | Qiaokou cohort | Caidian cohort |
| *Acinetobacter baumannii* | 17 | 9 |
| *Enterobacter cloacae* | 1 | 4 |
| *Enterococcus faecalis* | 9 | 3 |
| *Enterococcus faecium* | 7 | 3 |
| *Enterococcus gallinarum* | 1 | 0 |
| *Klebsiella pneumoniae* | 9 | 2 |
| *Listeria monocytogenes* | 4 | 3 |
| *Moraxella osloensis* | 1 | 4 |
| *Neisseria meningitidis* | 3 | 2 |
| *Pseudomonas aeruginosa* | 1 | 0 |
| *Pseudomonas antarctica* | 1 | 0 |
| *Pseudomonas stutzeri* | 1 | 0 |
| *Staphylococcus aureus* | 9 | 2 |
| *Staphylococcus capitis* | 7 | 9 |
| *Staphylococcus epidermidis* | 3 | 2 |
| *Streptococcus parasanguinis* | 1 | 0 |
| *Staphylococcus haemolyticus* | 5 | 7 |
| *Staphylococcus hominis* | 9 | 6 |
| *Staphylococcus kloosii* | 1 | 1 |
| *Streptococcus mitis* | 1 | 0 |
| *Staphylococcus pasteuri* | 1 | 0 |
| *Streptococcus pneumoniae* | 0 | 1 |
| *Streptococcus salivarius* | 1 | 0 |
| *Staphylococcus sciuri* | 0 | 2 |
| *Staphylococcus cohnii* | 0 | 1 |
| *Streptococcus suis* | 0 | 1 |
| *Streptococcus viridans* | 2 | 4 |
| *Staphylococcus warneri* | 3 | 0 |
|  | | |
