# Supplementary material for: Diagnostic Model for Discrimination Between Tuberculous Meningitis and Bacterial Meningitis
Source: Front Immunol. 2021 Nov 12;12:731876. doi: 10.3389/fimmu.2021.731876 (PMC8632769; doi:10.3389/fimmu.2021.731876)
Supplement: Supplementary file 2 [file Table_2.docx]

| **Supplementary Table 2. The results of logistic regression analysis** | | | | | |
| --- | --- | --- | --- | --- | --- |
| Variable | B | S.E | Wald | df | Exp (B) |
| TBAg/PHA ratio | 11.512 | 2.173 | 28.072 | 1 | 99869.15 |
| CSF chlorine | -0.149 | 0.04 | 14.177 | 1 | 0.861 |
| CSF nucleated cell count | -0.001 | 0.001 | 3.077 | 1 | 0.999 |
| CSF lymphocyte proportion | 0.041 | 0.01 | 16.475 | 1 | 1.042 |
| Constant | 13.677 | 4.375 | 9.775 | 1 | 870920 |
| TBAg, tuberculosis antigen; PHA, phytohaemagglutinin; CSF, cerebrospinal fluid. | | | | | |
